# Inequality in workplace support for various types of precarious workers compared with permanent workers in Japan: A cross‐sectional study

**DOI:** 10.1002/1348-9585.12431

**Published:** 2023-10-13

**Authors:** Kosuke Sakai, Tomohisa Nagata, Kiminori Odagami, Nuri Purwito Adi, Masako Nagata, Koji Mori

**Affiliations:** ^1^ Department of Occupational Health Practice and Management, Institute of Industrial Ecological Sciences University of Occupational and Environmental Health Japan Kitakyushu Japan; ^2^ Department of Community Medicine, Faculty of Medicine Universitas Indonesia Jakarta Indonesia; ^3^ Department of Occupational Medicine, School of Medicine University of Occupational and Environmental Health Japan Kitakyushu Japan

**Keywords:** contract worker, precarious worker, temporary worker, workplace support

## Abstract

**Objectives:**

The purpose of this study was to examine, by sex, whether precarious workers in Japan receive less support in the workplace than permanent workers.

**Methods:**

We conducted a cross‐sectional study using an online questionnaire in 2022. We stratified participants by sex and performed modified Poisson regression analysis. The outcomes were support from supervisors, co‐workers, occupational health professionals, and no one. Adjusted prevalence ratios (aPR) were calculated for contract workers, part‐time workers, and dispatched workers, using permanent workers as reference.

**Results:**

This study had 21 047 participants. For men, 87.9% were permanent workers; for women, 50.7% were permanent workers and 37.3% were part‐time workers. For workplace support, 47.5% of men and 45.2% of women selected superiors; 41.8% of men and 50.5% of women selected colleagues; 16.8% of men and 6.2% of women selected occupational health professionals. Female contract workers were less likely to receive support from their supervisors (aPR 0.88) or co‐workers (aPR 0.89). Male part‐time workers were less likely to be supported by their co‐workers (aPR 0.86). Dispatched workers were less likely to be supported by their supervisors (men aPR 0.71, women aPR 0.84) and co‐workers (men aPR 0.73, women aPR 0.77). Part‐time and dispatched workers were less likely to receive support from occupational health professionals.

**Conclusions:**

Precarious workers could get less workplace support than permanent workers. This may contribute to occupational health problems with precarious workers.

## 1 INTRODUCTION

The number of precarious workers is increasing in countries all over the world.^1^ This trend has been reported in the United States and European countries,^2^, ^3^ and also in Japan, where approximately 40% of workers are currently in precarious employment.^4^, ^5^

Previous studies in Japan have identified three types of precarious workers: contract workers, part‐time workers, and dispatched workers.^6^, ^7^ Contract workers are defined as workers working under fixed‐term contracts for professional employment or reemployment after retirement. Part‐time workers are defined as workers whose working hours are shorter than those of permanent workers, who work more than 40 hours per week. Dispatched workers have two contractual arrangements. First, they have an employment contract with one company (dispatching company) and they work under the supervision of another company (receiving company). There are differences between men and women in the background of precarious employment in Japan.^8^ Men tend to keep their careers as permanent workers, so there are many precarious workers who could not continue to work as permanent workers for various negative reasons. Women, on the contrary, tend to become precariously employed because they seek flexibility in working styles to care for their children and parents, so there are many precarious workers who chose to be precariously employed by themselves.^9^

In many workplaces, as the number of precarious workers increases, workers with various types of employment status are required to work with permanent workers, who are directly employed and have no limitation on their contract period. There may also be obvious disparities in pay and treatment among workers with different types of employment contract. It is therefore likely that precarious workers will face a variety of difficulties in working with permanent workers in the same workplace.^10^, ^11^

Previous studies have shown that it is beneficial for workers' long‐term health and safety to receive support in the workplace from supervisors, co‐workers, and occupational physicians.^12^ Supervisory coaching and support from co‐workers were reported to increase work engagement, characterized by vigor, dedication, and absorption in work.^13^ Higher workplace social capital (trust, norms, and networks) has been found to decrease mental health problems and the occurrence of workplace accidents.^14^, ^15^ Occupational physicians can advise workers on improving the work environment and their health.^16^, ^17^ Workplace support is therefore important for the health and safety of all workers.^18^ However, not all workers receive adequate support in the workplace.^19^

Precarious workers could be less supported than permanent workers in the workplace. They are often employed to cover temporary labor shortages and so are vulnerable to unemployment due to economic crises such as the Lehman Shock or the COVID‐19 pandemic.^20^ They may therefore change workplaces quite quickly and are repeatedly expected to build relationships with new co‐workers in different workplaces. This vulnerable position and repeated workplace changes could make precarious workers less likely than permanent workers to receive support in the workplace. In the United States, precarious workers, particularly if they are from racial or ethnic minorities or are immigrants, have been reported to be exposed to job insecurity and accident hazards.^21^ This suggests that they are not being adequately supported in the workplace. However, workplace support for precarious workers is likely to vary with national labor policy, laws, and occupational health system. As mentioned above, there are three types of precarious employment in Japan, and these types of employment should be considered and analyzed separately in research. Previous studies about workplace support for precarious workers in Japan have been limited to studies targeting specific populations or dealing with all types of precarious employment.^22^, ^23^ The aim of this study was therefore to examine, by sex stratification, whether the three types of precarious workers get less workplace support than permanent workers, and whether support varies by type of employment, using an online questionnaire.

## 2 MATERIALS AND METHODS

### 2.1 Study design and participants

We conducted a cross‐sectional study using data from a self‐administered questionnaire survey. The study was part of the W2S‐Ohpm study, run by a research group from the University of Occupational and Environmental Health, Japan.

We set the inclusion criterion for this study as those who were working at the time of response and conducted stratified sampling according to the sex, age, and geographical distribution of the national statistical data in February 2022.^24^ Our target sample size was approximately 30 000 respondents. The survey was initiated by Rakuten Insight, Inc. (located in Tokyo, Japan). Participation was exclusive to those enrolled with Rakuten Insight, via an online survey platform. Due to the confidentiality policy of the company, we lacked data about the initial number of invitations for the survey. The respondents answered the questionnaire from February 28 to March 3, 2022. We received responses from 59 272 registered monitors who completed the preliminary screening questions and participated in the investigation. A subset of 29 997 of these respondents met the inclusion criteria. The study protocol has previously been reported in detail.^25^

As data cleaning, we implemented checks for consistency and completeness. When a discrepancy was identified in a response, participants were prompted via on‐screen alerts that their answers were not coherent. This was primarily directed toward individuals who inserted nonsensical characters or symbols in the open‐ended response field, such as “AAA” and “$&¥.” Furthermore, we dismissed responses from participants who completed the survey in an unusually short time span, considering such inputs as invalid. To purge the data of deceptive responses post factum, we established the exclusion parameters to include: extraordinarily high body weight (exceeding 300 kg); extreme height (above 250 cm); and patently false answers (for instance, respondents reporting zero days and hours of work, those claiming over 150 h of work in a week, those reporting more than 100 overtime hours weekly, and those claiming to cohabitate with 18 or more family members). In the data cleaning, we excluded 2304 respondents due to invalid responses.

After data cleaning, the study generated survey data from 27 693 respondents. Following the purposes of this study, we excluded foreign nationals (*n* = 169). We then removed respondents with employment types outside of permanent or precarious categories, such as self‐employed (*n* = 2208), executive officers (*n* = 1595), freelancers (*n* = 2226), and others (*n* = 1112). Japanese workers retire at age 65 and receive a pension, and we therefore also excluded workers over age 65 (*n* = 2341) to minimize bias. This left 21 047 participants in our study.

This study was approved by the Ethics Committee of the University of Occupational and Environmental Health, Japan (R3‐076).

### 2.2 Assessment of employment status

The participants selected the most appropriate employment status from the following options in the questionnaire: (a) self‐employed, (b) executive officer, (c) permanent worker, (d) part‐time worker, (e) dispatched worker, (f) contract worker, (g) freelancer, or (h) other. We excluded (a), (b), (g), and (h) from the analysis.

### 2.3 Assessment of support in the workplace

The next question asked: “Do you have anyone in your workplace that you can talk to about your current job, work life, or concerns about health issues?”. Participants were able to select one or more options from (a) supervisor, (b) co‐worker or subordinate, (c) occupational physician, (d) occupational nurse, (e) hygiene manager or hygiene promoter, etc., (f) external counselor or consultation desk contracted by the business establishment, (g) labor union, (h) others, and (i) no one. Precarious workers do not usually have subordinates, and we therefore interpreted the choice of (b) co‐worker or subordinate as getting support from a co‐worker.

### 2.4 Other variables

In the questionnaire, respondents were asked to identify their sex as men or women, as it is recorded on their birth certificate or family register. We collected socioeconomic, job‐related, and health‐related factors as covariates. Socioeconomic factors were age, sex, education level (junior high school, high school, vocational school, junior college/technical college, university, and graduate school), and marital history (currently married, divorced/bereaved, and unmarried). Job‐related factors were occupations classified by Japan Standard Industrial Classification (Rev. 13, October 2013), job position (manager and nonmanager), years at present job (numerical), and size of workplace (2–4, 5–9, 10–29, 30–49, 50–99, 100–499, 500–999, 1000–9999, and more than 10 000 workers in the workplace).^26^ The health‐related factors included medical history, body mass index (BMI), and subjective health status. We asked about cardiovascular disease, cancer, hypertension, and diabetes as pre‐existing or undertreatment diseases. BMI was calculated from the self‐reported height and weight. The question of subjective health status was “How is your current health?”. Participants chose from six options ranging from very good to very bad.

Considering the possibility that respondents working multiple jobs, we asked them to respond to the questions regarding workplace support, occupation, job position, years at present job, and size of workplace by informing them that the questions were about their main job.

### 2.5 Statistical analysis

We applied a Poisson regression model with a robust error variance, modified Poisson regression, to analyze the relationship between employment status and workplace support. We defined four outcomes, which were support from a supervisor, co‐worker or subordinate, occupational health professional (occupational physician or nurse), and no one. A modified Poisson regression was performed for each of these four outcomes, stratified by sex. The objective variable was set to 1 for each of the four outcomes selected in the question about workplace support and 0 otherwise. We calculated adjusted prevalence ratios (aPR), 95% confidence intervals (CI), and *p*‐values for contract workers, part‐time workers, and dispatched workers compared with permanent workers as references. *p*‐values were derived using a two‐sided test, with the threshold for significance defined at .05. Covariates included age, education, marital status, occupation, job position, years at present job, size of workplace, and subjective health status. All analyses used Stata (Stata Statistical Software: Release 16; StataCorp LLC, TX).

## 3 RESULTS

### 3.1 Characteristics of the participants

Characteristics of 21 047 participants (11 107 men and 9940 women) are shown in Table 1. Overall, 87.9% of men and 50.7% of women were permanent workers and 37.3% of women were part‐time workers. In total, 63.7% of men and 39.3% of women had a university degree or higher; 22.8% of men and 4.7% of women were managers, and 26.1% of men and 9.7% of women had been in their current employment status for more than 20 years. Overall, 48.5% of men and 45.2% of women said they could obtain support from a supervisor, 41.8% of men and 50.5% of women said a co‐worker, 16.2% and 6.2% an occupational physician, and 28.7% and 26.0% no one. Details on the characteristics of respondents classified by sex and employment status are presented in Table S1..

**TABLE 1:** Demographic characteristics of the study population.

	Men		Women	
	*N*	%	*N*	%
Total	11 107	100.0	9940	100.0
Employment status				
Permanent worker	9759	87.9	5042	50.7
Contract worker	653	5.9	790	7.9
Part‐time worker	556	5.0	3709	37.3
Dispatched worker	139	1.3	399	4.0
Age, years				
20–29	2068	18.6	1986	20.0
30–39	2457	22.1	2060	20.7
40–49	3037	27.3	2735	27.5
50–59	2486	22.4	2270	22.8
60–65	1059	9.5	889	8.9
Education				
Junior high school	147	1.3	126	1.3
High school	2433	21.9	2488	25.0
Vocational school	1074	9.7	1595	16.0
Junior college or technical college	378	3.4	1820	18.3
University	6038	54.4	3622	36.4
Graduate school	1037	9.3	289	2.9
Material status				
Currently married	6392	57.5	4937	49.7
Divorced or bereaved	1099	9.9	1671	16.8
Unmarried	3616	32.6	3332	33.5
Occupation				
Agriculture, forestry, fisheries, and mining	81	0.7	49	0.5
Construction	638	5.7	322	3.2
Manufacturing	2792	25.1	1039	10.5
Electricity, gas, heat supply, and water	232	2.1	81	0.8
Information and communications	799	7.2	329	3.3
Transport and postal activities	756	6.8	272	2.7
Wholesale and retail trade	895	8.1	1346	13.5
Finance and insurance	430	3.9	580	5.8
Real estate and goods rental and leasing	214	1.9	151	1.5
Scientific research, professional and technical services	270	2.4	182	1.8
Accommodation and hospitality	205	1.8	511	5.1
Living‐related and personal services, entertainment services	168	1.5	318	3.2
Education, learning support	535	4.8	712	7.2
Medical, healthcare, and welfare	931	8.4	2254	22.7
Compound services	116	1.0	84	0.8
Services, not included elsewhere	861	7.8	1002	10.1
Government, except elsewhere classified	978	8.8	418	4.2
Industries unable to classify	206	1.9	290	2.9
Job position				
Manager	2529	22.8	465	4.7
Nonmanager	8578	77.2	9475	95.3
Size of workplace (number of employees)				
2–4	424	3.8	725	7.3
5–9	760	6.8	1142	11.5
10–29	1791	16.1	2244	22.6
30–49	1033	9.3	1109	11.2
50–99	1484	13.4	1277	12.8
100–499	2680	24.1	1823	18.3
500–999	869	7.8	552	5.6
1000–9999	1558	14.0	815	8.2
10 000+	508	4.6	253	2.5
Years at present job				
<1	811	7.3	1223	12.3
1–4	2449	22.0	3191	32.1
5–9	2247	20.2	2412	24.3
10–19	2697	24.3	2148	21.6
20+	2903	26.1	966	9.7
Medical history				
Cardiovascular disease	320	2.9	114	1.1
Cancer	259	2.3	305	3.1
Hypertension	2016	18.2	814	8.2
Diabetes	871	7.8	306	3.1
Body mass index(kg/m^2^)				
<18.5	549	4.9	1824	18.4
18.5≤, <25	7563	68.1	6960	70.0
25≤, <30	2441	22.0	890	9.0
30≤	554	5.0	266	2.7
Subjective health status				
Very good	1179	10.6	830	8.4
Good	3544	31.9	3286	33.1
Moderately good	4191	37.7	3922	39.5
Moderately bad	1735	15.6	1491	15.0
Bad	345	3.1	303	3.0
Very bad	113	1.0	108	1.1
Support in the workplace (multiple selections possible)				
Supervisor	5385	48.5	4493	45.2
Co‐worker	4647	41.8	5020	50.5
Occupational physician	1799	16.2	614	6.2
Occupational nurse	755	6.8	383	3.9
Health manager or health promoter	224	2.0	66	0.7
Outside counselor	456	4.1	245	2.5
Labor union	483	4.3	196	2.0
Other	71	0.6	130	1.3
No one	3191	28.7	2588	26.0

### 3.2 Employment status and workplace support

Table 2 shows the relationship between employment status and workplace support. Regarding support from supervisors, temporary workers (men aPR 0.71, women aPR 0.84) received less support than permanent workers. Female contract workers got less support from their supervisors than female permanent workers (aPR 0.88). There was no statistically significant difference, but male part‐time workers tended to get more support than permanent workers from their supervisors (aPR 1.10, 95% CI 0.99–1.21, *p*‐value = .068). Regarding support from co‐workers, part‐time workers (men aPR 0.86), contract workers (women aPR 0.89), and dispatched workers (men aPR 0.73, women aPR 0.77) got less support than permanent workers. Regarding support from occupational health professionals, part‐time workers (men aPR 0.34, women aPR 0.45) and dispatched workers (women aPR 0.26) got less support than permanent workers.

**TABLE 2:** Relationship between employment status and workplace supports from supervisors, co‐workers, occupational health professionals, and no one.

	*N*			Modified Poisson regression^a^		
	Total	Supported^b^	%	aPR	95% CI	*p*‐value
Supervisor						
Men						
Permanent workers	9759	4810	49.3	Reference		
Contract workers	653	276	42.3	0.98	0.89–1.08	0.730
Part‐time workers	556	256	46.0	1.10	0.99–1.21	0.068
Dispatched workers	139	43	30.9	0.71	0.55–0.91	0.007
Women						
Permanent workers	5042	2451	48.6	Reference		
Contract workers	790	307	38.9	0.88	0.80–0.97	0.009
Part‐time workers	3709	1590	42.9	0.97	0.92–1.03	0.298
Dispatched workers	399	145	36.3	0.84	0.73–0.96	0.011
Co‐worker						
Men						
Permanent workers	9759	4223	43.3	Reference		
Contract workers	653	222	34.0	0.95	0.85–1.07	0.385
Part‐time workers	556	166	29.9	0.86	0.75–0.98	0.025
Dispatched workers	139	36	25.9	0.73	0.55–0.97	0.030
Women						
Permanent workers	5042	2642	52.4	Reference		
Contract workers	790	357	45.2	0.89	0.82–0.96	0.005
Part‐time workers	3709	1871	50.4	0.98	0.93–1.03	0.488
Dispatched workers	399	150	37.6	0.77	0.68–0.88	< 0.001
Occupational health professional						
Men						
Permanent workers	9759	2001	20.5	Reference		
Contract workers	653	107	16.4	0.96	0.80–1.14	0.626
Part‐time workers	556	18	3.2	0.34	0.22–0.54	< 0.001
Dispatched workers	139	11	7.9	0.57	0.32–1.01	0.053
Women						
Permanent workers	5042	645	12.8	Reference		
Contract workers	790	79	10.0	0.86	0.68–1.08	0.201
Part‐time workers	3709	132	3.6	0.45	0.36–0.56	< 0.001
Dispatched workers	399	12	3.0	0.26	0.15–0.46	< 0.001
No one						
Men						
Permanent workers	9759	2669	27.3	Reference		
Contract workers	653	229	35.1	1.02	0.90–1.14	0.787
Part‐time workers	556	228	41.0	1.03	0.92–1.16	0.588
Dispatched workers	139	65	46.8	1.31	1.09–1.58	0.004
Women						
Permanent workers	5042	1201	23.8	0.00		
Contract workers	790	246	31.1	1.19	1.05–1.34	0.004
Part‐time workers	3709	983	26.5	1.03	0.94–1.12	0.551
Dispatched workers	399	158	39.6	1.41	1.23–1.62	< 0.001

Abbreviations: aPR, adjusted prevalence ratio; CI, confidence interval.

^a^ Adjusted for age, education, marital status, occupation, job position, years at present job, size of workplace, and subjective health status.

^b^ Number of respondents who selected supervisor, co‐worker, occupational health professional, or no one by sex and employment status in response to the multiple‐choice question, “Do you have anyone in your workplace that you can talk to about your current job, work life, or concerns about health issues?” For example, 4810 out of 9759 male permanent workers selected their supervisor in this question.

Dispatched workers were more likely than permanent workers to say that they had no one to provide support (men aPR 1.31, women aPR 1.41). Female contract workers were also more likely than female permanent workers to say that they had no one to provide support (aPR 1.19).

## 4 DISCUSSION

We analyzed the workplace support available to different types of workers in a cross‐sectional study. We found that precarious workers were less likely than permanent workers to be able to access workplace support in some situations. We assessed the differences for contract, part‐time, and dispatched workers. First, female contract workers did not get as much support from their supervisors and co‐workers as their permanent peers. Second, male part‐time workers did not get as much support from their co‐workers as male permanent workers. Third, dispatched workers obtained less workplace support than permanent workers from their supervisors, co‐workers, and occupational health professionals. By adjusting for possible socioeconomic, job‐related, and health‐related confounding factors, we were able to analyze the relationship between the employment situation itself and workplace support.

Female contract workers got less support than permanent workers from their supervisors and co‐workers and less support overall. In contrast, among men, there was no difference in workplace support between contract workers and permanent workers. There have been some reports of health problems among female contract workers. One study analyzed the relationship between precarious employment and depressive symptoms in Japan and found that female contract workers were more likely than permanent female workers to be depressed.^27^ In another study on cancer and employment among Japanese workers, contract workers were found to be more vulnerable to losing their jobs than permanent workers when they had breast cancer.^28^ These problems may be associated with the lack of workplace support for female contract workers. Workplace support should be strengthened to ensure that female contract workers can continue to work in good health.

Male part‐time workers were less likely than male permanent workers to report getting support from their co‐workers. Male part‐time workers are in a minority, especially compared with their female counterparts.^24^ Japan has a traditional social norm that men work to finance the household and women do the housework and childcare.^8^ Male part‐time workers often have a different working background from female part‐timers, who are married and work to supplement household income while still carrying out childcare and housework.^29^ A previous Japanese study reported that male part‐time workers were more likely to be depressed than male permanent workers.^27^ This finding may be associated with the lack of workplace support for male part‐time workers. We found, however, that the adjusted prevalence ratio for support from supervisors for part‐time male workers was 1.10 (95% CI 0.99–1.21, *p* = .068). These workers may therefore obtain their support from their supervisors, who are mostly men, rather than from their co‐workers.

Dispatched workers got less support than permanent workers from either supervisors or co‐workers. In fact, the results showed that dispatched workers got very little support in the workplace compared with permanent workers. We considered three reasons why this might be the case. First, the results may be affected by organizational structure. Dispatched workers have an employment contract with a dispatching company but work for a receiving company. In these circumstances, there may be confusion about whether to consult with the dispatching or receiving company. Second, they are often in a precarious situation. Previously, dispatched workers have been laid off in large numbers during recessions.^4^, ^30^ Seeking support from supervisors might therefore be interpreted negatively, marking them as “troublemakers” to be first in line for firing. Third, dispatched workers tend to change workplaces frequently, because the law limits the duration of dispatch to the same workplace to a maximum of 3 years.^31^ Within this legal system, unless the receiving company allows the dispatched workers to be employed directly, they must be moved to a different workplace. If they change workplaces, they will have to build new relationships. This puts them in a situation where they do not know anyone in the workplace, making it hard to obtain support from co‐workers.

Part‐time workers and dispatched workers did not receive support from occupational health professionals. The Occupational Health and Safety Law mandates that employers must appoint an occupational physician if they have 50 or more regular workers in a workplace.^32^ In other words, it means that employers with less than 50 workers do not have to appoint an occupational physician. Part‐time workers often work in small workplaces, such as stores and offices, so they probably do not get the support of occupational health professionals because they are not close to occupational health professionals. Temporary workers tended not to receive support from occupational health professionals, although no significant difference was observed among men (aPR 0.57, 95% CI 0.32–1.01). It would be difficult for dispatched workers to meet with occupational physicians from the dispatching company, who are responsible for providing their basic healthcare support. Even if dispatched workers seek support from the occupational health physician, these physicians are not familiar with every dispatched worker's workplace and therefore cannot provide appropriate advice. Japan's Industrial Safety and Health Act sets out that the responsibility for occupational health and safety of dispatched workers lies with the dispatching company. However, when this responsibility cannot be fulfilled by the dispatching company, for example, in establishing a safe work environment and preventing exposure to hazards, it is transferred to the receiving company. The complexity of this position makes it difficult for dispatched workers to get adequate occupational health support.^33^, ^34^ Dispatched workers are more frequently injured on the job and more likely to experience back pain and other similar conditions.^35^ It is therefore necessary to improve the current system so that dispatched workers can access occupational health services.

This study had some limitations. First, there are limitations regarding the validity and reliability of the measurement of employment status and workplace support. Respondents provided a subjective assessment of their employment status. To confirm the employment status responses, we calculated the mean and standard division (SD) of weekly working hours per status. For permanent workers, the mean was 39.2 h (SD 15.6), contract workers, 34.2 h (SD 13.7), part‐time workers, 23.3 h (SD 14.4), and temporary workers, 34.7 h (SD 14.5). Part‐time workers reported relatively shorter working hours. Additionally, the question about workplace support was asked using a multiple‐choice design, and the responses could therefore be affected by order bias. Many respondents selected their supervisors and co‐workers or subordinates, which were among the top few options. However, fewer respondents selected occupational physician or nurse, which were later in the list. In future studies, we would like to use an objective method of surveying employment status and workplace support.

Second, there might have been some selection bias. Participants were selected from people registered with an Internet‐based survey company, and they might therefore have higher digital literacy. Additionally, those who prefer not to participate in Internet surveys were not included in the target population. This would not be enough bias to have a significant impact on the results. This is because the distribution of employment status in this study was very similar to that in government statistics for the same period. These statistics show that among men aged 20–64 years old, there were 22.4 million permanent workers (85.0%), 1.5 million contract workers (5.7%), 2.0 million part‐time workers (7.7%), and 0.4 million dispatched workers (1.6%). Among women in the same age group, there were 11.9 million permanent workers (51.1%), 1.64 million contract workers (7.0%), 9.0 million part‐time workers (38.6%), and 0.78 million dispatched workers (3.3%).^24^

Third, it is not possible to identify a causal relationship. Given the cross‐sectional nature of this research, it only found associations between precarious employment and workplace support. It might be the case that precarious employment stems from deficient communication skills required to seek appropriate support. Further longitudinal studies should be planned to examine whether people who have become precariously employed fail to get support in the workplace.

To the best of our knowledge, this study is the first to identify the relationship between types of precarious workers and workplace support in Japan. We were able to adjust for various confounding factors using an online survey database. The study may therefore provide some insights into the complex situation of workplace support for three types of precarious workers.

## 5 PRACTICAL IMPLICATIONS

The results of this study highlight two problems to address to improve occupational health and safety for precarious workers. First, the circumstances that make it difficult for contract workers and part‐time workers to obtain support in the workplace should be improved. To increase workplace retention and achieve business continuity, it may be helpful to strengthen support for male part‐time workers and female contract workers in particular. Second, support for dispatched workers should be strengthened. The responsibilities of the dispatching and receiving company should be clearly defined, and dispatched workers should be supported appropriately by occupational health professionals. Some previous studies confirmed that precarious workers can continue to work in good health if they receive adequate support at their workplaces.^36^, ^37^ It is very important for businesses and the government to create a system that allows precarious workers to obtain appropriate workplace support.

## 6 CONCLUSIONS

Precarious workers had more difficulty than permanent workers in obtaining workplace support. The study revealed that barriers to receiving support from supervisors, co‐workers, and occupational health professionals varied by sex and type of precarious employment. Among contract, part‐time, and dispatched workers, the study found that dispatched workers were particularly lacking in workplace support when compared to permanent workers. These findings suggest that it is important to improve the occupational health system to better accommodate the needs of precarious workers.

## AUTHOR CONTRIBUTIONS

TN, KO, and KM contributed to study design; TN, KO, NA, MN, and KM contributed to data collection; KS and TN contributed to data analysis; KS contributed to drafting the manuscript; all authors have reviewed and approved the final manuscript.

## FUNDING INFORMATION

This study was supported and partly funded by a research grant from the University of Occupational and Environmental Health, Japan (no grant number); Japanese Ministry of Health, Labour and Welfare (210401‐01 and 20JA1005); JSPS KAKENHI (JP22K10543 and JP19K19471); Collabo‐Health study group (no grant number), TIS Inc. (no grant number), HASEKO Corporation (no grant number), DAIDO LIFE INSURANCE COMPANY (no grant number), and Hitachi Systems, Ltd. (no grant number). The funders were not involved in the study design, collection, analysis, interpretation of data, the writing of this article, or the decision to submit it for publication.

## CONFLICT OF INTEREST STATEMENT

The authors declare no conflicts of interest associated with this manuscript. TN reports a research grant from TIS Inc. and personal fees from BackTech Inc., EWEL Inc., and Sompo Health Support Inc., outside the submitted work. KM reports research grants from DAIDO LIFE INSURANCE COMPANY, Komatsu Ltd., and HASEKO Corporation; scholarship grants from AORC, BackTech Inc., DAIDO LIFE INSURANCE COMPANY, EWEL Inc., iSEQ.Inc., JMA Research Institute Inc., MEDIVA Inc., SMS Co., Ltd., Sompo Health Support Inc., and T‐PEC CORPORATION; and personal fees from BackTech Inc. and Sompo Health Support Inc., outside the submitted work.

## INFORMED CONSENT

Informed consent was obtained via the survey form on the website.

## REGISTRY AND THE REGISTRATION NO. OF THE STUDY/TRIAL

N/A.

## ANIMAL STUDIES

N/A.

## Supporting information


Table S1.

## Data Availability

The data that support the findings of this study are available on request from the corresponding author. The data are not publicly available due to privacy requirements or ethical restrictions.
