# Supplementary material for: Inequality in workplace support for various types of precarious workers compared with permanent workers in Japan: A cross‐sectional study
Source: J Occup Health. 2023 Oct 13;65(1):e12431. doi: 10.1002/1348-9585.12431 (PMC10576158; doi:10.1002/1348-9585.12431)
Supplement: Supplementary file 1 — Table S1. [file JOH2-65-e12431-s001.pdf]

Table S1. Demographic characteristics by sex and employment status

	Men									Women									
	Permanent		Contract		Part-time		Dispatched		Total	Permanent		Contract		Part-time		Dispatched		Total
	N	%	N	%	N	%	N	%	N	N	%	N	%	N	%	N	%	N
Total	9,759	87.9	653	5.9	556	5.0	139	1.3	11,107	5,042	50.7	790	7.9	3,709	37.3	399	4.0	9,940
Age, years																		
20–29	1,873	90.6	67	3.2	113	5.5	15	0.7	2,068	1,491	75.1	115	5.8	323	16.3	57	2.9	1,986
30–39	2,267	92.3	52	2.1	110	4.5	28	1.1	2,457	1,243	60.3	135	6.6	595	28.9	87	4.2	2,060
40–49	2,783	91.6	101	3.3	113	3.7	40	1.3	3,037	1,293	47.3	215	7.9	1,094	40.0	133	4.9	2,735
50–59	2,237	90.0	127	5.1	85	3.4	37	1.5	2,486	820	36.1	222	9.8	1,124	49.5	104	4.6	2,270
60–65	599	56.6	306	28.9	135	12.7	19	1.8	1,059	195	21.9	103	11.6	573	64.5	18	2.0	889
Education																		
Junior high school	103	70.1	19	12.9	18	12.2	7	4.8	147	31	24.6	7	5.6	84	66.7	4	3.2	126
High school	1,963	80.7	189	7.8	221	9.1	60	2.5	2,433	922	37.1	203	8.2	1,250	50.2	113	4.5	2,488
Vocational school	923	85.9	73	6.8	60	5.6	18	1.7	1,074	789	49.5	127	8.0	611	38.3	68	4.3	1,595
Junior college or technical college	321	84.9	36	9.5	17	4.5	4	1.1	378	723	39.7	165	9.1	852	46.8	80	4.4	1,820
University	5,471	90.6	300	5.0	223	3.7	44	0.7	6,038	2,369	65.4	264	7.3	863	23.8	126	3.5	3,622
Graduate school	978	94.3	36	3.5	17	1.6	6	0.6	1,037	208	72.0	24	8.3	49	17.0	8	2.8	289
Material status																		
Currently married	5,903	92.3	323	5.1	136	2.1	30	0.5	6,392	1,953	39.6	324	6.6	2,529	51.2	131	2.7	4,937
Divorced or bereaved	934	85.0	85	7.7	62	5.6	18	1.6	1,099	799	47.8	177	10.6	606	36.3	89	5.3	1,671
Unmarried	2,922	80.8	245	6.8	358	9.9	91	2.5	3,616	2,290	68.7	289	8.7	574	17.2	179	5.4	3,332
Medical history																		
Cardiovascular disease	265	82.8	30	9.4	19	5.9	6	1.9	320	67	46.5	17	11.8	58	40.3	2	1.4	144
Cancer	216	83.4	20	7.7	19	7.3	4	1.5	259	136	44.6	38	12.5	119	39.0	12	3.9	305
Hypertension	1,627	80.7	216	10.7	139	6.9	34	1.7	2,016	293	36.0	81	10.0	412	50.6	28	3.4	814
Diabetes	711	81.6	90	10.3	57	6.5	13	1.5	871	128	41.8	28	9.2	134	43.8	16	5.2	306
Body mass index(kg/m^2)																		
<18.5	449	81.8	31	5.6	51	9.3	18	3.3	549	954	52.3	133	7.3	662	36.3	75	4.1	1,824
18.5≤, <25	6,691	88.5	435	5.8	353	4.7	84	1.1	7,563	3,629	52.1	559	8.0	2,508	36.0	264	3.8	6,960
25≤, <30	2,153	88.2	158	6.5	106	4.3	24	1.0	2,441	347	39.0	72	8.1	424	47.6	47	5.3	890
30≤	466	84.1	29	5.2	46	8.3	13	2.3	554	112	42.1	26	9.8	115	43.2	13	4.9	266
Subjective health status																		
Very good	1,063	90.2	44	3.7	57	4.8	15	1.3	1,179	471	56.7	62	7.5	266	32.0	31	3.7	830
Good	3,171	89.5	203	5.7	143	4.0	27	0.8	3,544	1,682	51.2	258	7.9	1,216	37.0	130	4.0	3,286
Moderately good	3,675	87.7	262	6.3	193	4.6	61	1.5	4,191	1,949	49.7	320	8.2	1,499	38.2	154	3.9	3,922
Moderately bad	1,465	84.4	120	6.9	124	7.1	26	1.5	1,735	743	49.8	111	7.4	568	38.1	69	4.6	1,491
Bad	291	84.3	20	5.8	26	7.5	8	2.3	345	143	47.2	31	10.2	117	38.6	12	4.0	303
Very bad	94	83.2	4	3.5	13	11.5	2	1.8	113	54	50.0	8	7.4	43	39.8	3	2.8	108
Support in the workplace (multiple selections possible)																		
Supervisor	4,810	89.3	276	5.1	256	4.8	43	0.8	5,385	2,451	54.6	307	6.8	1,590	35.4	145	3.2	4,493
Co-worker	4,223	90.9	222	4.8	166	3.6	36	0.8	4,647	2,642	52.6	357	7.1	1,871	37.3	150	3.0	5,020
Occupational physician	1,696	94.3	89	4.9	8	0.4	6	0.3	1,799	479	78.0	55	9.0	74	12.1	6	1.0	614
Occupational nurse	690	91.4	47	6.2	11	1.5	7	0.9	755	272	71.0	35	9.1	70	18.3	6	1.6	383
Health manager or health promoter	204	91.1	17	7.6	1	0.4	2	0.9	224	48	72.7	1	1.5	16	24.2	1	1.5	66
Outside counselor	410	89.9	35	7.7	8	1.8	3	0.7	456	150	61.2	25	10.2	54	22.0	16	6.5	245
Labor union	457	94.6	17	3.5	7	1.4	2	0.4	483	136	69.4	18	9.2	42	21.4	0	0.0	196
Other	58	81.7	4	5.6	5	7.0	4	5.6	71	51	39.2	11	8.5	50	38.5	18	13.8	130
No one	2,669	83.6	229	7.2	228	7.1	65	2.0	3,191	1,201	46.4	246	9.5	983	38.0	158	6.1	2,588

The numbers in the table represent the count of individuals.
